# A toolkit enabling efficient, scalable and reproducible gene tagging in trypanosomatids

**DOI:** 10.1098/rsob.140197

**Published:** 2015-01-07

**Authors:** Samuel Dean, Jack Sunter, Richard J. Wheeler, Ian Hodkinson, Eva Gluenz, Keith Gull

**Affiliations:** 1 Sir William Dunn School of Pathology, University of Oxford, South Parks Road, Oxford OX1 3RE, UK; 2 Department of Computing, Imperial College London, South Kensington Campus, London SW7 2AZ, UK

**Keywords:** trypanosomatid, molecular tools, tagging, fusion PCR, homologous recombination, optimization

## Abstract

One of the first steps in understanding a protein's function is to determine its localization; however, the methods for localizing proteins in some systems have not kept pace with the developments in other fields, creating a bottleneck in the analysis of the large datasets that are generated in the post-genomic era. To address this, we developed tools for tagging proteins in trypanosomatids. We made a plasmid that, when coupled with long primer PCR, can be used to produce transgenes at their endogenous loci encoding proteins tagged at either terminus or within the protein coding sequence. This system can also be used to generate deletion mutants to investigate the function of different protein domains. We show that the length of homology required for successful integration precluded long primer PCR tagging in *Leishmania mexicana*. Hence, we developed plasmids and a fusion PCR approach to create gene tagging amplicons with sufficiently long homologous regions for targeted integration, suitable for use in trypanosomatids with less efficient homologous recombination than *Trypanosoma brucei*. Importantly, we have automated the primer design, developed universal PCR conditions and optimized the workflow to make this system reliable, efficient and scalable such that whole genome tagging is now an achievable goal.

## 2. Introduction

Trypanosomatids are unicellular eukaryotic parasites that include important human pathogens such as *Trypanosoma brucei*, the aetiological agent of human sleeping sickness, and *Leishmania* spp., which cause visceral and cutaneous infections. High quality, well-annotated genomes are available for trypanosomatids, and molecular tools have been created for both *T. brucei* and *Leishmania* spp*.* to facilitate gene knockouts and ectopic gene expression [1–3]. Moreover, an effective inducible expression system has been developed for *T. brucei* [4], this being further augmented with RNAi knockdown [5], which allows in depth analysis of gene function.

Advances in DNA and RNA sequencing, mass spectrometry and bioinformatics have facilitated high-throughput approaches such as genome-wide RNAi screens [6–8], proteomes [9–12] and transcriptomes [13–16]. These advances result in large datasets, highlighting numerous genes of interest; yet often these genes have minimal annotation in the genome databases. Hence, there is a need for large-scale validation of protein localization and function in these datasets.

Studies of protein localization provide important assistance with the more difficult elucidation of protein function. While antibody approaches offer the opportunity to detect the wild-type protein, gene tagging methods, while having a number of caveats, are the only ones capable of facilitating large cohort analysis in realistic timeframes. Plasmids that enable the tagging of a gene of interest in *T. brucei* at its endogenous locus [17] rely on traditional restriction enzyme cloning methods, which can be time-consuming and technically challenging. However, trypanosomes require only 50 nucleotides of identity for targeted homologous recombination [18] which can be incorporated into a PCR primer for long primer PCR tagging [19,20]. This method requires a plasmid that contains a tag and resistance gene separated by an appropriate intergenic sequence that provides mRNA processing signals. The tag and resistance gene are then amplified using long primers that contain a short region of identity to the plasmid and a longer region (50–100 nucleotides) of homology to the appropriate region of the gene locus to be tagged. Unfortunately, application of this approach has been limited in the *T. brucei* research community, perhaps due to lack of reproducibility from gene to gene and the need for multiple, nested PCRs.

To overcome the limitations of the existing methods and develop a protocol that is reliable, cheap and scalable to hundreds of genes, we focused on developing the long primer PCR tagging method. Hence, we constructed plasmids that act as a template for long primer PCR, supporting tagging of *T. brucei* proteins at either terminus or embedded within the protein. This approach can also be used to generate deletion mutants to investigate the function of different protein domains. This is a powerful workflow that will be invaluable in determining protein localization and function and for validating new and existing proteomes.

Molecular tools for *Leishmania* spp*.* are available [1,2,21,22] but not as well developed as those for *T. brucei*, and here we used two approaches to create a new and improved toolkit for *Leishmania mexicana*. Firstly, a set of plasmids that facilitate the endogenous tagging of proteins in *L. mexicana* with a variety of different tags was developed. These plasmids are of a modular design that allows the rapid exchange of components, such as the drug resistance gene and the tag, with the trypanosome pEnT series of vectors [17]. The long primer PCR endogenous gene tagging approach did not work in *L. mexicana* due to lower recombination efficiency with short homology lengths [23]. We showed that transfection efficiency in *L. mexicana* was proportional to homology length between the target gene and the tagging construct and that reliable transfection required longer homologous regions than can be generated by long primer PCR. Therefore, we developed a second approach using a fusion PCR method, enabling the rapid production of endogenous gene tagging amplicons with sufficiently long homologous regions for efficient transfection without any cloning steps.

## 3. Results

### 3.1. Gene tagging in *Trypanosoma brucei*

#### 3.1.1. Development of the PCR only tagging series of plasmids

The PCR only tagging (pPOT) series of plasmids were designed to allow rapid and scalable endogenous tagging of target genes. Hence, the DNA sequence encoding a tag (e.g. eYFP) and a resistance cassette are amplified from pPOT using long primers that incorporate a 5′ overhang of 80 nucleotides of homology to the target gene and its adjacent untranslated region (UTR). The amplicon (referred to as the long primer PCR amplicon) is then used to transfect *T. brucei* and recombines such that the tag is fused to the target gene with recombinant cell lines selected using the appropriate drug.

**Figure 1.**
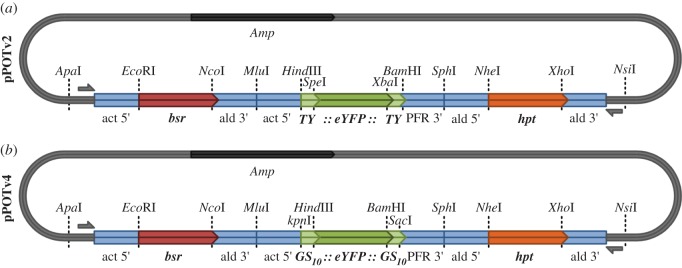
Plasmids for PCR only tagging of genes in *T. brucei* or *Leishmania*. Plasmid map of (*a*) pPOTv2 and (*b*) pPOTv4 with useful unique restriction enzyme sites indicated. pPOTv1 and pPOTv3 are development versions of the pPOT series of plasmids that are less modular than pPOTv2 and v4 and are therefore not shown here.

**Figure 2.**
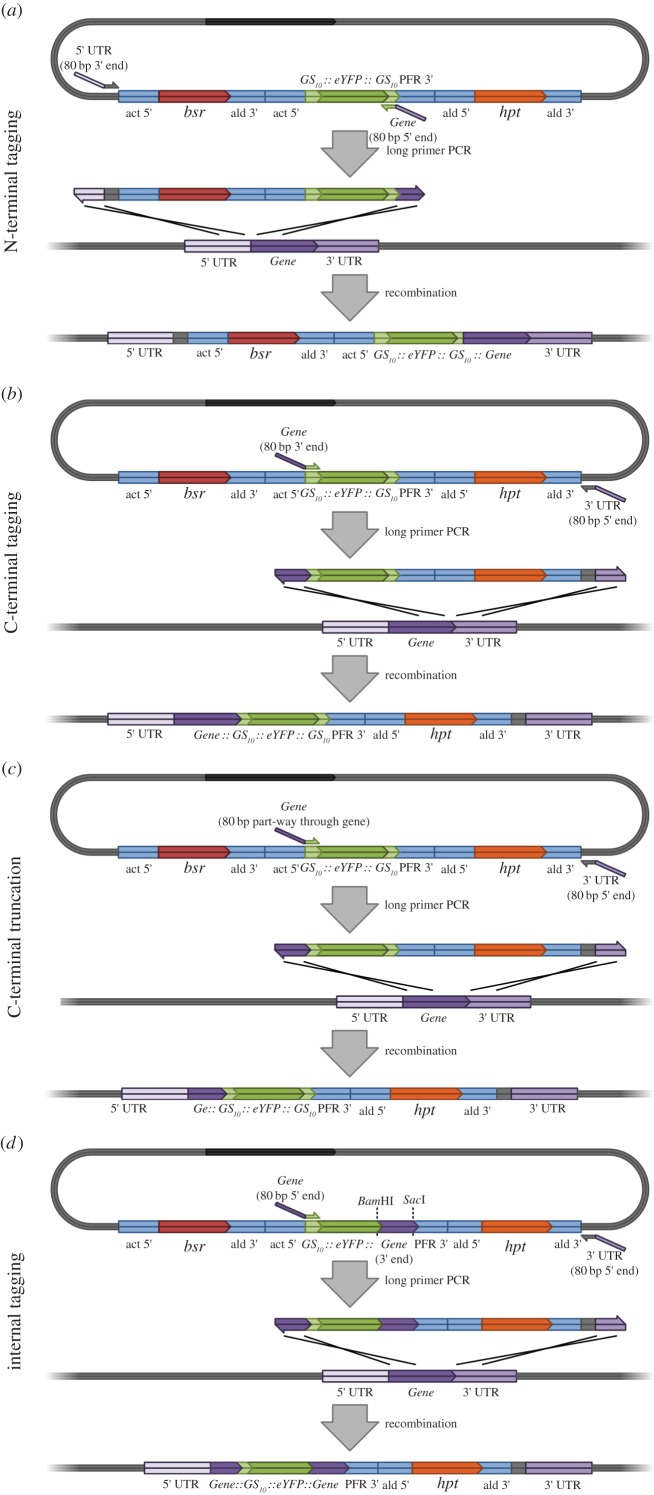
Long primer PCR tagging and deletion mutagenesis using pPOTv4. (*a*) N-terminal tagging. (*b*) C-terminal tagging. (*c*) Creating a C-terminal deletion mutant. (*d*) Internal tagging towards the N-terminal end of the gene (using a modified pPOTv4 template).

For general purpose tagging where adequate antibody reagents are available (for either the protein or the tag), we recommend using pPOTv4 (figure 1). pPOTv4 contains the following, ordered 5′ to 3′: a unique forward primer binding site, *actin* 5′ UTR, *blasticidin S deaminase* (*bsr*) gene, *aldolase* 3′ UTR, *actin* 5′ UTR, *GS(10)::eYFP::GS(10)* (each *GS* linker contains a unique primer binding site), *PFR2* 3′ UTR, *aldolase* 5′ UTR, *hygromycin phosphotransferase* (*hpt*) gene, *aldolase* 3′ UTR, a unique reverse primer binding site. pPOTv4 is entirely modular, such that each component is flanked by unique restriction enzyme sites and can be replaced using traditional cloning methods. pPOTv4 is designed to support the production of both N, C and internal tagging amplicons (figure 2). The inclusion of a glycine–serine linker may assist the folding of fusion proteins with challenging characteristics (such as slow folding or hydrophobic proteins). The *eYFP* tag between the *GS* linker sequence can be changed using the *Hind*III *Bam*HI restriction enzyme sites. The careful placement of unique primer annealing sequences means that tags and resistance genes can be changed in pPOTv4 without redesigning the tagging primers (see the electronic supplementary material, plasmids available upon request). This means that the same long primer pair can be used to target a gene with any tag using any resistance drug for selection, depending on how the pPOTv4 template is modified. This has significant cost advantages and further increases the flexibility of the system.

In cases where additional antibody detection reagents would be useful (e.g. double labelling), we recommend using pPOTv2. pPOTv2 is identical to pPOTv4, except that the sequence encoding the *GS::eYFP::GS* is replaced with *Ty::eYFP::Ty*. The Ty epitope can be detected by the BB2 monoclonal antibody [24]. In addition, the *eYFP* tag can be changed using *Spe*I *Xba*I restriction enzyme sites for other markers, such as the APEX electron microscopy marker and the halo tag (see the electronic supplementary material, plasmids available upon request). The *GS* linker sequence is not present in pPOTv2, so must be encoded on the tagging primer if a linker is required on the fusion protein. As with pPOTv4, pPOTv2 is entirely modular and can be modified with different drug resistance genes and tags; however, because one of the amplification primers anneals to the genetic tag, if the tag is changed then this primer must be re-designed.

#### 3.1.2. Primer design enables amino, carboxyl and internal tagging in addition to deletion analysis

Primers to endogenously tag the N-terminus of a target protein using pPOTv4 are designed as follows. From 5′ to 3′, the forward primer consists of the last 80 nucleotides of the 5′ UTR of the gene of interest followed by the 20 nucleotide 5′ pPOTv4 primer binding sequence. From 5′ to 3′, the reverse primer consists of the first 80 nucleotides of the target open reading frame (ORF) in reverse complement followed by the last 18 nucleotides of the 3′ GS linker sequence without the stop codon in reverse complement. Similarly, the primers to endogenously tag the C-terminus of the target protein using pPOTv4 are designed as follows. From 5′ to 3′, the forward primer consists of the last 80 nucleotides of the target ORF without the stop codon followed by the first 18 nucleotides of the 5′ GS linker sequence. From 5′ to 3′, the reverse primer consists of the first 80 nucleotides of the 3′ UTR of the target gene in reverse complement followed by the 20 nucleotide 3′ pPOTv4 primer binding sequence in reverse complement. The primers are then used in a PCR with pPOTv4 as template to produce the long primer PCR amplicon.

Making a deletion mutant is a simple and effective method of investigating the contribution of individual domains and specific amino acid sequences to protein localization and function. pPOTv2/v4 can be used to generate C-terminal deletion mutants by replacing the C-terminal part of the protein with an eYFP (figure 2*c*). Primers to do this using pPOTv4 are designed as follows. From 5′ to 3′, the forward primer consists of the last 80 nucleotides of the truncated target ORF in frame with the first 18 nucleotides of the 5′ GS linker sequence. From 5′ to 3′, the reverse primer consists of the first 80 nucleotides of the 3′ UTR of the target gene in reverse complement followed by the 20 nucleotide 3′ pPOTv4 primer binding sequence in reverse complement. Similarly, primers for N-terminal deletion mutants using pPOTv4 are designed as follows. From 5′ to 3′, the forward primer consists of the last 80 nucleotides of the 5′ UTR of the gene of interest followed by the 20 nucleotide 5′ pPOTv4 primer binding sequence. From 5′ to 3′, the reverse primer consists of the first 80 nucleotides of the truncated target ORF in reverse complement in frame with the last 18 nucleotides of the 3′ GS linker without the stop codon in reverse complement.

For certain proteins, the addition of an N- or C-terminal tag can disrupt proper folding, processing or localization of the fusion protein; for instance, GPI-anchored proteins have a signal peptide on the N-terminus, and a GPI-anchor addition site on the C-terminus, meaning that neither terminus is suitable for tagging. In these circumstances, pPOTv2/4 can be used to insert the tag within the target ORF using the long primer PCR methodology (figure 2*d*). For example, in order to insert the tag downstream of the signal peptide of a target protein, the signal peptide sequence is cloned into pPOTv4 as a *KpnI Hind*III fragment in-frame with the *eYFP*, replacing the 5′ GS linker sequence. The long primers are then designed as follows. From 5′ to 3′, the forward primer consists of the last 80 nucleotides of the 5′ UTR of the gene of interest followed by the first 20 nucleotides of the 5′ pPOTv4 primer binding sequence. From 5′ to 3′, the reverse primer consists of the 80 nucleotides of the target ORF after the predicted signal peptide in reverse complement followed by the last 18 nucleotides of the 3′ GS linker sequence in reverse complement without the stop codon. After transfection, the resulting cell line has the eYFP inserted between the signal peptide and the rest of the protein. Details of all the DNA sequences for primer design are given in table 1.

**Table 1.** The DNA sequences recommended for designing pPOT tagging and deletion mutant primers. The DNA sequence corresponding to the gene locus is always 5′ to that of the sequence that anneals to the pPOT template.

targeting strategy	forward or reverse primer	target locus sequence	pPOT annealing sequence
N-terminal tag using pPOTv4 (*GS::eYFP::GS*)	forward	the last 80 nucleotides of the target gene's 5′UTR	gtataatgcagacctgctgc
	reverse	the first 80 nucleotides of the target gene's ORF in reverse complement	actacccgatcctgatcc
C-terminal tag using pPOTv4 (*GS::eYFP::GS*)	forward	the last 80 nucleotides of the target ORF (excluding the stop codon)	ggttctggtagtggttcc
	reverse	the first 80 nucleotides of the target gene's 3′UTR in reverse complement	ccaatttgagagacctgtgc
N-terminal tag using pPOTv2 (*Ty::eYFP::Ty*)	forward	the last 80 nucleotides of the target gene's 5′UTR	gtataatgcagacctgctgc
	reverse	the first 80 nucleotides of the target gene's ORF in reverse complement	cttgtacagctcgtccatgc
C-terminal tag using pPOTv2 (*Ty::eYFP::Ty*)	forward	the last 80 nucleotides of the target gene's ORF (excluding the stop codon)	actagtgtgagcaagg
	reverse	the first 80 nucleotides of the target gene's 3′UTR in reverse complement	ccaatttgagagacctgtgc
N-terminal deletion using pPOTv4	forward	the last 80 nucleotides of the target gene's 5′UTR	gtataatgcagacctgctgc
	reverse	80 nucleotides corresponding to the 5′ end of the truncation in reverse complement	actacccgatcctgatcc
C-terminal deletion using pPOTv4	forward	80 nucleotides corresponding to the 3′ end of the truncation	ggttctggtagtggttcc
	reverse	the first 80 nucleotides of the target gene's 3′UTR in reverse complement	ccaatttgagagacctgtgc
internal tag. Note that the pPOTv4 template must be modified to contain the tag adjacent to either the 5′ or 3′ end of the target ORF	forward	the last 80 nucleotides of the target gene's 5′UTR	gtataatgcagacctgctgc
	reverse	80 nucleotides corresponding to the target ORF sequence downstream of that included in the pPOT template	actacccgatcctgatcc

#### 3.1.3. Development of universal PCR conditions

**Figure 3.**
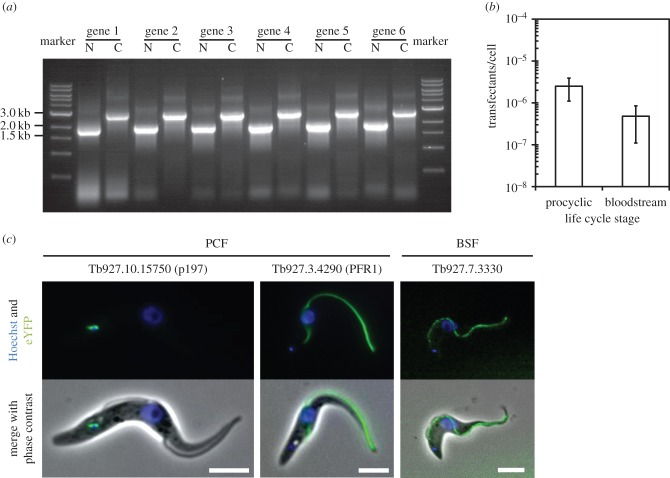
Examples of long primer PCR tagging using pPOT. (*a*) Example long primer PCRs to tag the N- and C-terminus of six different genes using pPOT. (*b*) Tagging efficiencies for procyclic and BSF *T. brucei* (error bars represent standard deviation)*.* (*c*) Epifluorescence of live *T. brucei* cells expressing proteins endogenously tagged at their C-terminus with eYFP generated using long primer PCR tagging. Scale bars, 5 µm.

We optimized the long primer PCR using primer pairs designed to tag eight different genes on the N-terminus, and one primer pair with identical template annealing sequences but no 5′ overhang as a control (see electronic supplementary material, figure S1*a*). We varied the annealing temperature, the type of polymerase, the use of dimethyl sulfoxide (DMSO) as a supplement, and whether the polymerase was added as ‘hotstart’. We found that the presence of the 80 nucleotide 5′ overhang inhibited the PCR under most of the conditions tested, causing either non-specific bands or the reaction to fail entirely. However, by using Expand HiFi polymerase, 2% DMSO, 65°C annealing and adding the polymerase in the first 94°C denaturation step, we were able to identify universal long primer PCR conditions; using the fully optimized PCR conditions, we have obtained yields of approximately 4 µg per 50 µl reaction from more than 99% of primer pairs (*n* > 200). An example set of PCRs to tag six different proteins on both the N- and C-terminus is shown in figure 3*a*. For a detailed PCR protocol, see the electronic supplementary material. Development of these universal PCR conditions means that large cohorts of genes can be tagged without the need to optimize the PCR on a gene-by-gene basis.

#### 3.1.4. Optimizing trypanosome transfections

In insect procyclic form (PCF) *T. brucei*, we found that transfection of 50 µl of un-purified long primer PCR gave a transfection efficiency of approximately 2.5 × 10^−6^ transfectants per cell; this transfection efficiency is sufficiently high to ensure that transfectants are reliably produced (figure 3*b*). Examples of PCF cells with proteins that have been tagged using long primer PCR amplicon are shown in figure 3*c*. Depending upon the target gene, the percentage of correctly integrated long primer PCR amplicons (as judged by the percentage of cells expressing the correctly localized eYFP-tagged protein in a non-clonal population) usually ranged from 90 to 100%.

In bloodstream form (BSF) *T. brucei*, we were unable to obtain transfectants with long primer PCR amplicons using conventional electroporation; this is probably due to the inherently lower transfection efficiency of BSF cells. For this reason, we switched to the Amaxa nucleofection system, which has been shown to increase transfection efficiency [25]. Using the Amaxa Nucleofector II and the T-cell system, we were able to obtain resistant cell lines with an average efficiency of approximately 4.8 × 10^−7^ transfectants per cell (figure 3*b*). An examples of a BSF cell with a protein tagged using long primer PCR amplicons is shown in figure 3*c*. A detailed transfection protocol for both PCF and BSF cells is in the electronic supplementary material.

### 3.2. New gene tagging tools in *Leishmania mexicana*

#### 3.2.1. Development of the *Leishmania* endogenous tagging plasmids

**Figure 4.**
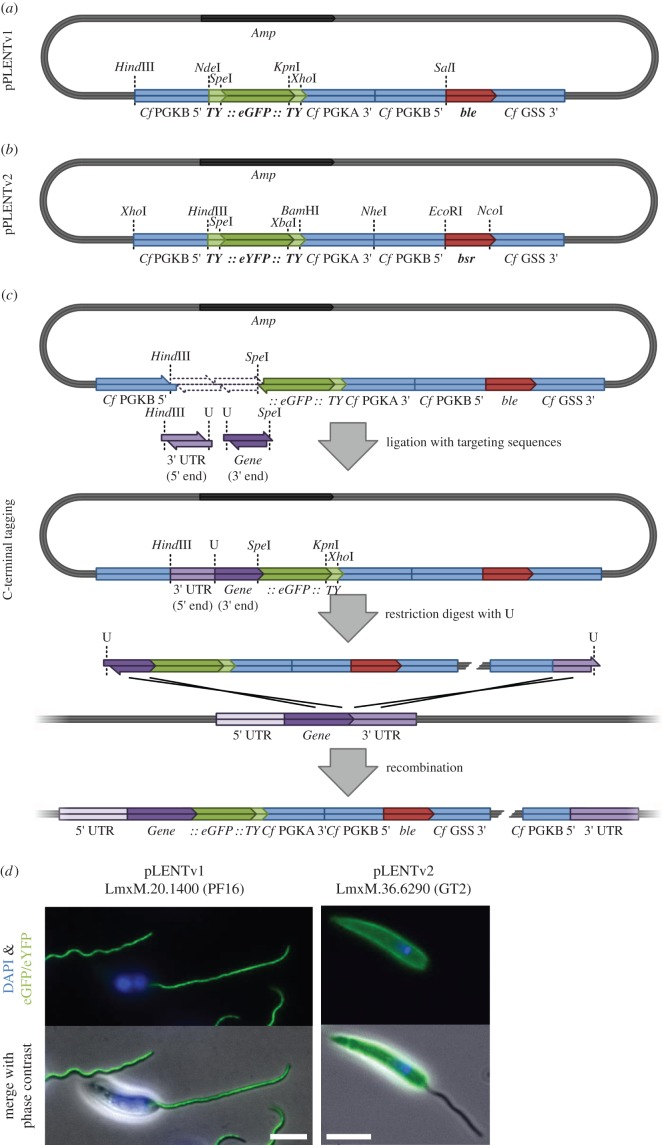
Plasmid-based endogenous gene tagging in *Leishmania* using pLENTv1 and pLENTv2 plasmids. (*a*) Plasmid map of pLENTv1 with useful restriction enzyme sites indicated and (*b*) plasmid map of pLENTv2-YB with useful unique restriction enzyme sites indicated. (*c*) Workflow to create and transfect a plasmid to endogenously tag a gene at its C-terminus. (*d*) Epifluorescence of live *L. mexicana* cells expressing PF16 endogenously tagged at its C-terminus with eGFP using pLENTv1 and glucose transporter 2 endogenously tagged at its C-terminus with eYFP using pLENTv2-YB. Scale bars, 5 µm.

We developed the *Leishmania* endogenous tagging (pLENT) series of plasmids to facilitate N- and C-terminal endogenous gene tagging using standard restriction enzyme cloning. The pLENT plasmids were derived from the pNUS plasmid series which was developed to allow the constitutive expression of transgenes in *Crithidia fasciculata* and *Leishmania* spp. using *Crithidia* mRNA processing signals [1]. Hence, from 5′ to 3′ pLENT contains: *C. fasciculata phosphoglycerate kinase* (*PGK*) *B* 5′ UTR, a *Ty::eGFP::Ty* tag, the *PGK A* 3′ UTR, the *PGK B* 5′ UTR, the *bleomycin-binding protein* (*ble*) gene, the 3′ UTR from the *C. fasciculata glutathionylspermidine synthetase* gene (figure 4*a*). A first generation plasmid was developed called pLENTv1, which was used to confirm the plasmid series would function as anticipated. For general purpose tagging, we recommend using pLENTv2 (figure 4), which has restriction enzyme sites compatible with the pEnT series of plasmids [17] (figure 4*a*). The tag can be changed as a *Spe*I *Xba*I fragment, the resistance gene can be changed as an *Eco*RI *Nco*I fragment and the 3′ UTR of *PGKA* can be removed with *Bam*HI *Nhe*I and replaced with another 3′ UTR to regulate the mRNA levels of the tagged gene.

To tag a gene at the C-terminus using pLENTv2, firstly, 300–500 nucleotides of the 3′ end of the ORF of interest (excluding the stop codon) is amplified using primers that contain a unique restriction enzyme site (for linearization of the final plasmid) and a *Spe*I site on the forward and reverse PCR primers, respectively. Secondly, 300–500 nucleotides immediately downstream of the ORF of interest is amplified using primers that contain *Hind*III and the linearization restriction enzyme site on the forward and reverse primers, respectively. The two amplified fragments are digested and ligated into *Hind*III *Spe*I cut pLENTv2. The resulting plasmid is linearized using the unique restriction site and then used for transfecting *L. mexicana*. A similar approach can be used for N-terminal tagging using pLENTv2 (see the electronic supplementary material).

Using pLENT, we have tagged more than 30 genes in *L. mexicana* including glucose transporter 2 (LmxM.6.6290) (figure 4*d*). Derivatives of pLENTv2 have been produced (table 2), with different tags and different resistance genes. Here, the endogenous gene tagging using the pLENT plasmid series has been performed in *L. mexicana* but the plasmid is suitable for use in any *Leishmania* species and related species including *C. fasciculata*.

**Table 2.** pLENTv2 derivatives showing the different tags and resistance genes.

resistance	tag		
	eYFP	dTomFP	CFP
Bsr (blasticidin)	pLENTv2-YB	pLENTv2-TB	pLENTv2-CB
Neo (G418)	pLENTv2-YN	pLENTv2-TN	pLENTv2-CN
Pac (puromycin)	pLENTv2-YP	pLENTv2-TP	pLENTv2-CP

#### 3.2.2. Optimizing *Leishmania* transfections

In order to develop PCR-based tools for genetic modification of *L. mexicana*, we first optimized cloning on 96-well plates to determine the transfection efficiency. After trialling a number of different conditions, we found wild-type *L. mexicana* grew from low culture densities on 96-well plates at 28°C with 5% CO_2_ in M199 supplemented with adenine and biopterin (MM199). These additives had previously been described for cloning *L. mexicana* on agar plates [3,26]. Positive wells seeded at less than 0.1 cells per well typically reached 1.0 × 10^6^ cells per well in 6–7 days, indicating a doubling time of approximately 7 h, similar to larger culture volumes in M199 [27]. For a detailed protocol of growth and counting of individual clones, see the electronic supplementary material.

**Figure 5.**
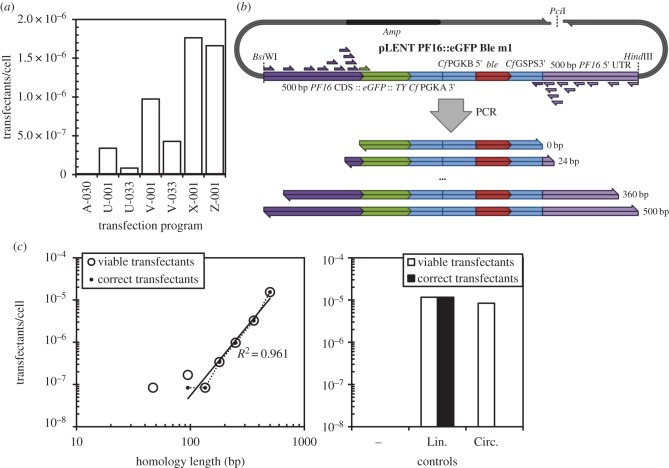
Dependency of homologous recombination on length of homology in *L. mexicana*. (*a*) Optimization of transfection efficiency with the Amaxa Nucleofector II and the T-cell system. A total of 1.2 × 10^7^ cells were transfected with 4.5 µg linearized pLENTv1 PF16::eGFP, and the number of drug-resistant transfectants were determined by transferring the entire culture to 96-well plates 8 h after transfection, with drug selection. Program X-001 has previously been used for BSF *T. brucei* [25] and U-033 has previously been used for *Leishmania* [28]. (*b*) Schematic of the PCR-based method of generating recombination substrates from pLENT PF16::eYFP Ble m1 (linearized with *Pci*I) with different lengths of homology for the endogenous tagging of PF16 with eYFP. In addition, circular plasmid, linearized plasmid (with 500 bp homology) and PCR with no primers (as a negative control) were prepared. (*c*) Dependency of viable transfection and correct recombination efficiencies on homology length, relative to the controls of vector linearized with *Bsi*WI and *Hind*III (Lin.), circular vector (Circ.) and PCR with no primers (−). A total of 1.2 × 10^7^ cells were transfected with 2 pmol DNA amplicon (1.00 × 10^5^ DNA molecules per cell), or the equivalent volume of the no primer negative control PCR.

We then optimized transfection using the Amaxa Nucleofector II with the T-cell system, counting the number of clones obtained to determine transfection efficiency (figure 5*a*). We found X-001 was the most efficient program and we therefore used it for all further *L. mexicana* transfections. For a detailed protocol for transfection, see the electronic supplementary material. We correlated the transfection efficiency of *Leishmania* with the length of homology in the transfected DNA by using a pLENT-derived PF16 eYFP tagging vector as a template in a PCR to generate products with lengths of homology between 0 and 500 bp (figure 5*b*). The number of viable transfectants and the number of transfectants with the expected fluorescence signal from PF16::eGFP (representing correct integration) were determined as described above.

The transfection efficiency of the circular plasmid, linearized plasmid and PCR product with 500 bp homology were approximately equal (1 × 10^−5^ transfectants per cell) and all transfectants from the linearized plasmid showed correct integration. Transfection using PCR products with lengths of homology between 0 and 500 bp showed that the transfection rate was linearly dependent on homology length (figure 5*c*). The vast majority of viable transfectants showed correct integration, with occasional mis-integration (as assessed by lack of expected fluorescence signal) at the shortest homology lengths. This showed that the shortest feasible homology length required for a recombination substrate was approximately 100 bp, with practical transfection efficiencies (more than 1 × 10^−6^ transfectants per cell) achieved with homology lengths above 300 bp (figure 5*c*). This result shows that long primer PCR is not a viable approach for gene tagging in *L. mexicana* and explains previous unsuccessful PCR-based gene tagging attempts [19].

#### 3.2.3. Endogenous gene tagging using a fusion PCR approach

The pLENT plasmids generate endogenous gene tagging constructs through traditional restriction enzyme cloning, which limits the number of targets that can be rapidly tagged as each plasmid takes days to make. To increase the throughput of gene tagging in *L. mexicana* and overcome homology length requirements, a fusion PCR approach was developed that allows the rapid creation of gene tagging amplicons in a single day.

**Figure 6.**
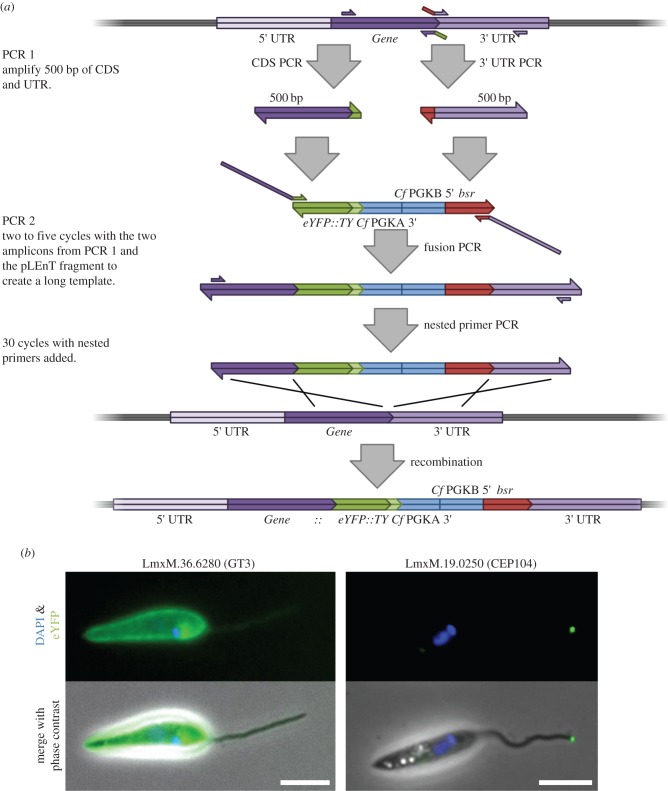
Fusion PCR tagging of *Leishmania* genes using pLENTv2. (*a*) The creation of an endogenous gene tagging amplicon by fusion PCR. (*b*) Epifluorescence of live *L. mexicana* expressing a C-terminally tagged glucose transporter and an N-terminally tagged CEP104 generated by fusion PCR. Scale bars, 5 µm.

To create a C-terminal tagging amplicon using pLENTv2-YB via the fusion PCR method, two rounds of PCR amplification are required (figure 6*a*). In the first round two fragments are amplified from genomic DNA. First, 500 bp of the 3′ end of the gene of interest (excluding the stop codon) is amplified. A 30 bp region of homology to the 5′ end of the *eYFP* gene is added to the 5′ end of the reverse primer for the target gene. Second, 500 bp of the 3′ intergenic region downstream of the STOP codon is amplified. Thirty base pairs of homology to the 3′ end of the *bsr* gene is added to the 5′ end of the forward primer for the target intergenic region.

The second round of PCR combined three pieces of DNA, the two 500 bp fragments amplified in the previous round and the central region from the pLENTv2-YB plasmid containing the *eYFP::Ty* gene followed by the *C. fasciculata PGKA* and *B* intergenic region and the *bsr* gene, which has been released by a *Spe*I *Nco*I digest and subsequent purification. These three fragments were combined in a fusion PCR of only five cycles without any primers to create a long tagging amplicon that can then act as a template for a PCR using nested primers that anneal 20–30 bp from the 5′ end of the gene fragment and 20–30 bp from the 3′ end of the UTR fragment. The PCR with the nested primers results in the generation of an amplicon that consists of the 3′ end of the gene of interest fused to the *eYFP::Ty* gene followed by an intergenic sequence then the *bsr* gene with the 3′ UTR of the gene of interest; the amplicon is then purified and used for the transfection of *L. mexicana*. The fusion PCR approach produced a tagging amplicon identical to that which would be created following the linearization of a pLENT plasmid but without the bacterial backbone. A similar strategy was adopted for N-terminal tagging (see the electronic supplementary material); however, pLENTv2 is not suitable for N-terminal tagging so the pPOTv2 plasmid was used to provide the central portion for the second round of PCR, specifically the section released by a *Eco*RI *Xba*I digest to give the *bsr* gene followed by an intergenic sequence and then the *Ty::eYFP* gene. Using the PCR-based approach, we have now tagged approximately 25 genes in *L. mexicana* including glucose transporter 3 (LmxM.36.6280) and CEP104 (LmxM.19.0250) (figure 6*b*).

### 3.3. Automated generation of primer sequences

In order to make PCR tagging as rapid and scalable as possible, using both the long primer and fusion PCR approaches, we generated two tools for the automatic design of primers for a particular gene, which should also reduce the number of incorrectly designed primers. The first tool is focused on high-throughput tagging of *T. brucei* and was designed to include as much reference information about the gene as possible. A number of useful features are incorporated into this tool, such as customizable warnings, predictions of the accuracy of the ORF based on experimental data [15,16], the presence of important N- or C-terminal sequence features such as signal peptides and GPI-anchor addition sites and whether the amplicon may tag non-target genes due to the high degree of homology between the ORFs. This tool comes in the form of a Perl script (electronic supplementary material) such that the user inputs a list of accession numbers, the terminus to be tagged, and which pPOT version is to be used, and the output is the primer designs and associated warnings. The long primer sequences to target every annotated gene in the *T. brucei* 927 genome using either pPOTv2 or pPOTv4 are in the electronic supplementary material.

The second tool was focused on flexibility and is an Internet based tool (http://www.richardwheeler.net/dnatools). This can be used to generate primers for fusion or long primer PCR for any gene in seconds, using one of a selection of plasmids as the template DNA, any homology/primer length and any target primer annealing temperature desired. Unlike the Perl tool, the online tool dynamically loads sequence data from TriTrypDB/EuPathDB [29,30] and can be used to generate primers for any gene in any species currently indexed, or which is added in the future. It is, however, limited by the current accuracy of annotation in TriTrypDB and does not perform any tests based on transcriptomic or proteomic evidence for the targeted gene.

## 4. Discussion

The sensitivity of both proteomic and transcriptomic methods has increased substantially in the last 5–10 years; however, the tools to investigate the function of the genes of interest identified by these approaches have not kept pace. We focused on developing tools to enable rapid endogenous gene tagging in both *T. brucei* and *Leishmania* spp., which allows the localization of target proteins, hence providing insight into their function.

The high rate of homologous recombination in *T. brucei* coupled with the short region of homology required for specificity of integration [18] presents an opportunity to develop a fast and scalable protein tagging system. We have optimized the PCR conditions such that more than 99% of reactions produce enough long primer PCR amplicon for a successful transfection. Furthermore, we have developed tools and a highly optimized workflow to facilitate tagging hundreds, if not thousands, of genes. To date in our laboratory, as part of a few focused projects, we have tagged more than 400 genes. Hence, tagging every protein coding gene in the trypanosome genome is now a realistic proposition.

The long primer PCR approach can be used to modify the protein beyond the addition of a tag at either the N- or C-terminus. Modification of the pPOT plasmid allows the integration of a tag within the protein so that features such as a signal peptide are retained at the N-terminus of the target protein. Importantly, with careful design of the long primers, both N- and C-terminal deletion mutants of a target protein can be produced for a functional analysis.

The molecular tools in *Leishmania* spp. have lagged behind those developed for *T. brucei*. We concentrated on creating a set of tools and methods to enable efficient endogenous gene tagging in *L. mexicana*. Firstly, we developed a set of modular plasmids: pLENT, in which the components are interchangeable so any combination of tag or drug can be used. This makes these plasmids an excellent choice to use when introducing tags into previously modified cell lines that may already contain certain fluorescent proteins and resistance markers.

In our hands, and as previously reported [19], long primer PCR endogenous gene tagging was not successful in *Leishmania* and we showed that this was due to *Leishmania* requiring longer (more than 100 nucleotides) regions of homology for correctly targeted homologous recombination than is the case for *T. brucei*. Commercially available primers are generally limited to 100–120 nucleotides; longer primers are available but these are prohibitively expensive, although this is likely to change in the future.

A cloning-free method to produce gene knockout amplicons for *T. brucei* based on fusion PCR was recently published [31], showing that the method was effective and scalable. Here, we used the same approach to perform endogenous gene tagging much more rapidly than can be done by traditional restriction enzyme cloning, such that enough fusion PCR amplicon for transfection can be made within a day. As with the long primer PCR method in *T. brucei*, the fusion PCR system developed here can be used to produce protein truncations and to integrate the tag within the protein.

We have developed a set of modular plasmids and PCR methods that will accelerate the functional analysis of genes in the trypanosomatids and will open up unexplored experimental avenues of investigation. All reagents are available upon request.

## 5. Material and methods

*Trypanosoma brucei* procyclic cells of the strain SMOXP9 [32] were grown at 28°C in SDM-79 medium supplemented with 10% FCS. The cultures were maintained between 2 × 10^5^ and 2 × 10^7^ cells ml^−1^.

*Trypanosoma brucei* BSF cells of the strain SMOXB4 [32] were grown at 37°C in HMI-9 medium supplemented with 15% FCS. The cultures were maintained between 1 × 10^5^ and 2 × 10^6^ cells ml^−1^.

*Leishmania mexicana* promastigote form cells of the WHO strain MNYC/BZ/62/M379 were grown at 28°C in M199 supplemented with 10% FCS, haemin and HEPES. The cultures were maintained between 1 × 10^5^ and 2 × 10^7^ cells ml^−1^.

Cell densities were determined using a CASY Cell Counter.

Cycling and reaction conditions for PCR to generate the linear fragments of DNA for analysis of recombination rate in *L. mexicana* were performed as described for long primer PCR (see electronic supplementary material).

For epifluorescence microscopy of live cells expressing fluorescent fusion proteins, 5 × 10^6^ cells were harvested from culture by centrifugation, washed three times with PBS containing 500 ng µl^−1^ Hoechst 33342, resuspended in 100 µl of PBS containing 500 ng µl^−1^ Hoechst, and 15 µl was settled on a glass slide with a coverslip laid on top prior to imaging.

*Leishmania* were transfected using the Amaxa Nucleofector II T-cell system. Sterile DNA was prepared by either ethanol precipitation of cut plasmid DNA or by purification from a PCR using the Qiagen PCR purification kit in a sterile environment. The number of drug-resistant transfectants were determined by transferring the entire transfected culture to 96-well plates in MM199 (for *L. mexicana*) at 200 μl per well with the appropriate selection drugs 8 h following transfection. For more details, see the electronic supplementary material. After two weeks, the number of wells with visible growth, *m*, was counted and the corresponding number of transfectants, *n*, was calculated assuming random distribution among the wells according to the Poisson distribution
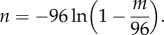


## Supplementary Material

Supplementary data

## Data accessibility

Final DNA sequences are uploaded in the electronic supplementary material.
