# Supplementary material for: A toolkit enabling efficient, scalable and reproducible gene tagging in trypanosomatids
Source: Open Biol. 2015 Jan 7;5(1):140197. doi: 10.1098/rsob.140197 (PMC4313374; doi:10.1098/rsob.140197)
Supplement: Supplementary data [file rsob140197supp1.zip › Supplementary data/Supp F1.pdf]

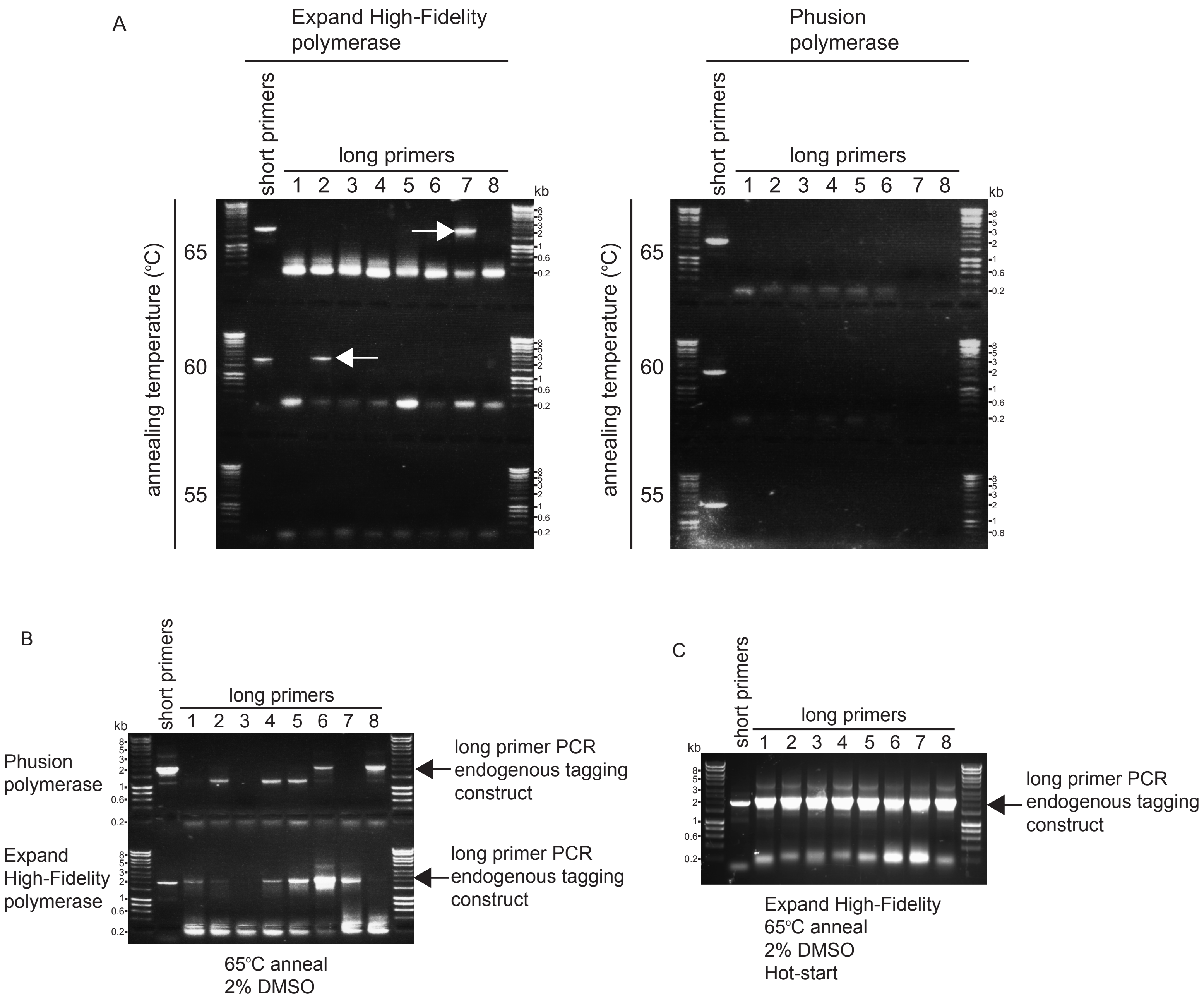

Supplementary Figure 1. Optimisation of long primer PCR.

A) The effect of different annealing temperatures and polymerases on the amplification of long primer PCR endogenous tagging construct.The PCR products were separated on an agarose gel stained with ethidium bromide. The white arrows indicate the expected product.

B) The effect of DMSO on the amplification of long primer PCR endogenous tagging construct. The PCR products were separated on an agarose gel stained with ethidium bromide. The black arrows indicate the expected products.

C) The effect of ‘hot-start’ on the amplification of long primer PCR endogenous tagging construct using Expand High-Fidelity polymerase. The PCR products were separated on an agarose gel stained with ethidium bromide. The black arrow indicates the expected products.
