# Supplementary material for: A toolkit enabling efficient, scalable and reproducible gene tagging in trypanosomatids
Source: Open Biol. 2015 Jan 7;5(1):140197. doi: 10.1098/rsob.140197 (PMC4313374; doi:10.1098/rsob.140197)
Supplement: Supplementary data [file rsob140197supp1.zip › Supplementary data/tagit1.0/Instructions for TAGit perl script.docx]

**Instructions for TAGit perl script**

**Notes**

TAGit automates the primer design for tagging 427 and 927 genes on either termini using pPOTv2 (Ty::YFP::Ty) or pPOTv4 (GS_(10)_::YFP::GS_(10)_).

The PCR_templates file can be modified to design primers for derivatives of pPOTv2 that contain tags other than Ty::YFP::Ty (see instructions in PCR_templates file, in the data folder)

TAGit will issue the following warnings if necessary:

- The gene model conflicts with either the Ochsenreiter or Cross experimental data
- The user tries to tag the N terminus and the gene is predicted to have an N terminal signal peptide
- The genome data in this region is bad and should not be trusted (i.e. contains any “n”s etc)
- It does not recognize the accession number
- If the primer that anneals to the ORF matches another, non-target gene, due to high degree of homology (=clashes). By default, up to 3 substitutions are allowed, but this can be increased or decreased as necessary. For example:
  - perl tagit.pl –dN Tb927.xx.xxxx (where N is the number of substitutions allowed).

The warnings files can be modified to issue custom warnings.

Given that members of labs often work on similar structures and biological mechanisms, it is inevitable that they will “bump” into each other and tag the same gene, thus duplicating work. This has been a particular issue when the number of genes tagged within the lab rises to the hundreds. To avoid this, we strongly suggest that when a new gene is tagged, it is added to the “Clash_warnings.txt” file. Tagit will then issue a warning when:

1. It is asked to design primers to any gene in this file
2. It is asked to design primers where the ORF annealing primer “clashes” with a gene in this file.

TAGit can use old accession numbers and will give you both the input accession and the new accession number in the output. However, it will use the new accession number in the unique primer name.

**Instructions for use**

**Notes**

More detailed instructions are found within the perl script (open in text edit or similar).

TAGit can be used in either batch mode, or for individual primer designs.

You will need the perl script itself and all the data files.

The data files should be saved inside the “Data files” folder, which itself should be in the same folder as the perl script (or you will have to tell the script where to find them)

**Primer design for individual genes:**

1. Open “Terminal”
2. Change directory to the folder containing the TAGit perl script
   1. Type “cd” and than drag the folder into the command line prompt. This should auto-fill the path to TAGit (if this doesn’t work, fill in the path manually)
   2. Press enter
3. Type: perl tagit.pl XYZ
   1. X = the version of pPOT currently in use (i.e. 2 or 4)
   2. Y = the terminus to be tagged. I.e. either “N”, “C” or “B” (both)
   3. Z = the accession number
   4. E.g. perl tagit.pl 4BTb927.1.2010
4. This will give the results in the command line, which can be pasted into excel.
5. Alternatively, append “ > results” to the command, and a separate file will be made called “results.xls” (or whatever you want to call it)
   1. E.g. perl tagit.pl 1BTb927.1.2010 > results.xls
6. Primer requests can be separated by spaces,
   1. E.g. perl tagit.pl 4BTb977.1.2010 2NTb927.1.2020 > results.xls

**Batch mode**

1. For larger numbers of genes it is easier to fill in the template. Hence fill in the below in the TAGit template file:
   1. Accession number: use the tritrypDB accession number
   2. PCR template: “2” or “4”)
   3. Tag terminus: “N”, “C”, or “B” (= amino, carboxyl or both)
2. Save the file in “Tab delimited text” format (e.g. primerinput.txt) in the same folder as TAGit
   1. NOTE – IF YOU SAVE AS AN EXCEL FILE IT WILL NOT WORK
3. The command should take the format:
   1. perl tagit.pl primerinput.txt > primerdesigns.xls
   2. note that the file extension “.txt” is usually hidden, but must be included in the command if it is part of the input file name

**Instructions for updating the data files**

Periodically, tritrypDB updates their databases. There are 4 files in the data folder that should be updated when there is a major update:

- Trypanosome: contains all the ORFs of the trypanosome genome. Necessary for both N and C terminal tagging
- Trypanosome-3’UTR: contains all the sequences downstream of the ORF, necessary for C terminal tagging
- Trypanosome-5’UTR: contains all the sequences upstream of the ORFs, necessary for N terminal tagging
- Accession number conversion: converts old TriTrypDB accession numbers into new ones (especially useful for analysis of older transcriptomics or proteomics data)

**Instructions for updating these files are as follows:**

1. go to: <http://tritrypdb.org/tritrypdb/>
2. On the tritryp homepage, perform the following search:
   1. New Search 🡪 Search for Genes 🡪 Text, IDs, Organism
3. Tick: “Trypanosoma brucei Lister strain 427” and “Trypanosoma brucei TREU927”
4. Click: Get Answer
5. Click: Download genes
6. Select: FASTA (sequence retrieval, configurable)
   1. Select: CDS
      1. Download as a text file, and save in the data folder as: Trypanosome
   2. Select: genomic
      1. Select: Translation start (ATG), -, 1
      2. Select: Translation start (ATG), -, 90
      3. Download as a text file, and save in the data folder as: Trypanosome-5’UTR
   3. Select: genomic
      1. Select: Translation Stop Codon, +, 1
      2. Select: Translation Stop Codon, +, 90
      3. Download as a text file, and save in the data folder as: Trypanosome-3’UTR
7. Select: Tab delimited (Excel)
   1. Click: clear all
   2. Tick: Previous ID(s)
   3. Download as text file, and save in data folder as: Accession number conversion
   4. NOTE – do not include the .txt extension
