# Supplementary material for: A toolkit enabling efficient, scalable and reproducible gene tagging in trypanosomatids
Source: Open Biol. 2015 Jan 7;5(1):140197. doi: 10.1098/rsob.140197 (PMC4313374; doi:10.1098/rsob.140197)
Supplement: Supplementary data [file rsob140197supp1.zip › Supplementary data/tagit1.0/tagitFAQ.pdf]

TagIT FAQ

version 0.95

June 2, 2014

What does TAGIT do? It computes primer sequences for tagging genes.

How do I run it? It can be run from a terminal program (e.g., `/Applications/Utilities/Terminal.app` on Macs). To run it, issue a command '`perl tagit.pl`' with any number of requests as parameters, separated by spaces. Example:

```
perl tagit.pl 1NTb927.8.970
perl tagit.pl pcr14NTb927.8.970 2CTb11.02.0230 2NTb11.02.0230
```

What does `1NTb927.8.970` mean? The 1 is the id of the desired PCR template. The N is the desired tag terminus (N or C). The rest is the desired gene accession number.

What if I want both N and C termini? Use 'B' as the terminus. Eg: `1BTb927.8.970`.

What if I have a lot of requests? You could put them in a file — a 'job file'. The format of each line should be:

```
Accession number△PCR template△Tag terminus
```

where △ is tab. Example:

```
Tb927.8.970△Templ. 61△N
```

Blank lines and lines starting `#` (comment lines) are ignored by the script. Files can be made and edited on Mac, PC, or Linux.

How do I run a job in a job file?

```
perl tagit.pl jobfile.txt
```

Warning: include any file extension (eg .txt), or the script won't find the file.

Can I mix command-line requests with job files? Yes. Example:

```
perl tagit.pl 1NTb927.8.970 jobfile1 2CTb11.02.0230 jobfile2
```

What files does the script use? See table 1.

Where should I put the files? The script assumes that the files `Trypanosome-5'UTR`, `Trypanosome`, `Trypanosome-3'UTR`, `Accession number conversion`, `PCR_templates`, and `Warnings.txt` are in a directory/folder called `data files` in the same directory as the script. If you want to put them somewhere else, just edit the first few lines of the script to point the relevant variables to the right files.

File name	Separation character	Line break character
Trypanosome-5'UTR	(machine generated)	Linux
Trypanosome	(machine generated)	Linux
Trypanosome-3'UTR	(machine generated)	Linux
Accession number conversion	(machine generated)	Linux
PCR_templates	comma	Mac/Windows/Linux
Warnings.txt	comma or tab	Mac/Windows/Linux
Job files (if used)	tab	Mac/Windows/Linux
Script output (if sent to a file)	tab	Linux

Table 1: the data files

Why tab-separated and comma-separated files? Yes, it's a bit of a hotch-potch. Files that are likely to be made with Excel etc use tabs to separate items, as this seems standard for exported spreadsheets. Files likely to be made by hand are comma-separated, as commas are more easily visible. The `Warnings.txt` file might be made by either method, so both commas and tabs are allowed in this file. Probably we should move to allowing both tabs and commas as separation characters in all files.

Why do some files require Linux line breaks? The data files `Trypanosome-5'UTR`, `Trypanosome`, `Trypanosome-3'UTR`, and the file `Accession number conversion` should have Unix/Linux style linebreaks, as they are large and the script would take a lot of time to sort them out if they were in other formats. They are machine generated and usually come with Linux line breaks anyway.

What's a line break? Generally, the character that's entered when you hit the return key. It's different on different systems. See `http://en.wikipedia.org/wiki/Newline`

Where are the PCR templates? In the file `PCR_templates`. The format of each line is comma-separated, with six items in the following order:

1. template id — a short easily-typed string
2. template name (full identifier)
3. sequence to be added to the forward primer for terminus N
4. sequence to be added to the reverse primer for terminus N
5. sequence to be added to the forward primer for terminus C
6. sequence to be added to the reverse primer for terminus C

Blank lines and lines starting `#` (comment lines) are ignored by the script. The file can be made and edited on Mac, Windows, or Linux.

What's a template id? Every PCR template should have its own unique id. It's supposed to be easy to type. It can be any string of text satisfying the following:

- At least one non-whitespace character
- Doesn't start with `#`
- No commas

- No spaces, if you want to use it in command-line requests (spaces are allowed in requests in job files)
- You can use `N`, `C` or `B`, but when using it in a command-line request you'll need to put a comma after the id — e.g., `myPCRtempl,NTb11.02.0230`.

Surrounding whitespace is ignored, so don't use any.

What's a template name? The full name of the PCR template. Example: `pPOTv2 blast Ty::mCherry::Ty hygro` (this is why we have template ids as well). Template names mustn't include commas (because commas are used as delimiters in the PCR template file). Other than that, pretty much anything is allowed.

What about the output? Output is to the terminal (STDOUT). The script first prints a header line — typically

```
Accession number△Old accession number△PCR template△Tag terminus△
For/Rev△Primer name△Primer sequence
```

where △ is tab. Subsequent lines follow this format. Example:

```
Tb09.160.0465 pPOTv2 blast Ty::mCherry::Ty hygro N F 2NTb09.160.0465F AGAAAC...
Tb09.160.0465 pPOTv2 blast Ty::mCherry::Ty hygro N R 2NTb09.160.0465R AGAAGC...
```

How can I write the output to a file? By standard unix/linux redirection. Just include a '>' parameter: `>file-name` to overwrite, `>>file-name` to append. Examples:

```
perl tagit.pl 1NTb927.8.970 >res.txt        [overwrites res.txt if exists]
perl tagit.pl >>myres.txt 1NTb927.8.970     [appends to myresults.txt]
perl tagit.pl jobfile1 2CTb11.02.0230 jobfile2 >results
perl tagit.pl jobfile3 >>oldresults jobfile4
```

What order are the results outputted in? Same as the order of requests received.

What happens if I enter the same request twice? You'll get two sets of identical results.

What if I enter an out-of-date accession number? The script tries to convert each entered accession number to an up-to-date equivalent, using the file `Accession number conversion`. An entered accession number will be converted if:

1. it cannot be found in the three main data files (the accession numbers in these files are assumed to be up to date), and
2. it occurs as an 'old' number in exactly one line of the file `Accession number conversion`

The entered number is then converted to the number appearing in the first column of the line of the file `Accession number conversion` found in (2). If a conversion occurred, the new accession number will appear in the leftmost column of the results, and the original accession number, as entered, will appear in column 2 (as per the header line).

What are warnings? The script sometimes appends one or more warnings to a result. The possible warnings are:

1. Any warnings found in the data files
2. Any warnings found in the file `Warnings.txt`
3. `Bad sequencing data` — when the nucleotide data contains `N` or other non-`ACGT` characters; the number of them is reported.
4. `Found only` *n* `<file> nucleotide(s)` — when fewer than 80 nucleotides were found.
5. `PCR template not found` — when the PCR template id does not match any line in the file `PCR_templates`.
6. `Accession number not found` — when the entered accession number is not found in the three main data files, and can't be updated.
7. `Accession number old and deprecated` — when the entered accession number is not found in the three main data files, and occurs as an 'old' number in more than one line of the file `Accession number conversion`. The accession number will not be updated. Because it is not in the main data files, it will generate an `Accession number not found` warning as well.
8. `Ambiguous updated accession number?` — the entered accession number was updated to a number that also occurs as an 'old' number in exactly one line of the file `Accession number conversion`.

What's the `Warnings.txt` file? It contains warnings and can be customised. The format of each line is comma- or tab-separated, as the user chooses, with three items in the following order:

1. accession number
2. terminus: `N`, `C`, or `B` (for both N and C). The warning message will appear on the results for the specified termini.
3. text of warning message

Blank lines and lines starting `#` (comment lines) are ignored by the script. The file can be made and edited on Mac, Windows, or Linux. The supplied (default) `Warnings.txt` file contains warnings compiled from the Cross and Ochsenreiter gene model files, and signal peptide warnings.

The script produced thousands of results. How can I tell if there are warnings? Look at the header line. It includes as many `Warning` headers as necessary. Whenever any warnings were generated, there'll be at least one `Warning` at the end of the header line.

Which files are tab-separated and which are comma-separated? I'm confused. See table 1.
